# Carnitine Serum Levels in Frail Older Subjects

**DOI:** 10.3390/nu12123887

**Published:** 2020-12-19

**Authors:** Giulia Malaguarnera, Vito Emanuele Catania, Claudia Bonfiglio, Gaetano Bertino, Enzo Vicari, Michele Malaguarnera

**Affiliations:** 1 Department of Biomedical and Biotechnological Science, University of Catania, 95123 Catania, Italy; giulia.malaguarnera@live.it (G.M.); michele.malaguarnera@gmail.com (M.M.); 2 Medical, Surgical Sciences and Advanced Technologies “G.F. Ingrassia”, University of Catania, 95123 Catania, Italy; 3 Medical Centre INPS, 25129 Catania, Italy; bonfiglio@gmail.com; 4 Department of Experimental and Clinical Medicine, University of Catania, 95123 Catania, Italy; gaetanobertinounict@gmail.com (G.B.); evicari@unict.it (E.V.)

**Keywords:** carnitine, frailty, biomarkers, physical activity, cognitive function, fatigue, exhaustion, mitochondria

## Abstract

Frailty is an expression that reconciles and condenses loss of autonomy, both physical and cognitive decline and a wide spectrum of adverse outcomes due to aging. The decrease in physical and cognitive activity is associated with altered mitochondrial function, and energy loss and consequently morbidity and mortality. In this cross-sectional study, we evaluated the carnitine levels in frailty status. The mean serum concentrations of total carnitine (TC) were lower in frail elderly subjects than in prefrail ones (*p* = 0.0006), higher in frail vs. robust subjects (*p* < 0.0001), and higher in prefrail vs. robust subjects (*p* < 0.0001). The mean serum concentrations of free carnitine (FC) were lower in frail elderly subjects than in prefrail ones (*p* < 0.0001), lower in frail vs. robust subjects (*p* < 0.0001) and lower in prefrail vs. robust subjects (*p* = 0.0009). The mean serum concentrations of acylcarnitine (AC) were higher in frail elderly subjects than in prefrail ones (*p* = 0.054) and were higher in pre-frail vs. robust subjects (*p* = 0.0022). The mean urine concentrations of TC were lower in frail elderly subjects than in prefrail ones (*p* < 0.05) and lower in frail vs. robust subjects (*p* < 0.0001). The mean urine concentrations of free carnitine were lower in frail elderly vs. robust subjects (*p* < 0.05). The mean urine concentrations of acyl carnitines were lower in frail elderly subjects than those in both prefrail (*p* < 0.0001) and robust subjects (*p* < 0.0001). Conclusion: high levels of carnitine may have a favorable effect on the functional status and may treat the frailty status in older subjects.

## 1. Introduction

Frailty is a geriatric syndrome characterized by a multidimensional, transitional state of increases vulnerability and loss of ability to adapt to exogenous or endogenous stress [1]. The prevalence of frailty ranges from 1.0% to 22.0% [2], but due to the aging of the population, this percentage will increase resulting in high rates of hospitalization. As a consequence, it will considerably increase the public health care costs [3]. Multiple factors that contribute to frailty include inflammation, neuromuscular dysfunction, endocrine dysregulation, immune dysfunction, abnormalities in energy metabolism and central nervous system failure in older people [4,5,6,7]. The decline in the functional capabilities is multifactorial and the dysregulated mitochondrial metabolism may be a root of age-related frailty. Mitochondria are involved in a variety of critical cell functions including oxidative energy production, programmed cell death, growth and redox signaling. Several reports showed that the treatment with carnitine may have an impact on mitochondrial biogenesis. In previous studies, we observed that oral administration of L-carnitine increases total muscular masses, restores physical and cognitive capacities, and reduces fatigue [8,9,10]. Carnitine is a quaternary ammonium cation, mainly synthesized in organs with a higher metabolic activity, such as kidney, liver, and brain. It can also be integrated through the consumption of animal products like meat, fish, poultry and milk [11]. The main function of carnitine is the transport of long chain fatty acid to the inner mitochondrial membrane, allowing the β-oxidation [12]. Other functions of carnitine include buffering of mitochondrial acyl-CoA/CoA ratio, the oxidation of branched amino-acids and a protective effect on the endogenous antioxidant defense system [13,14]. Most studies have suggested that carnitine may play some role in muscle weakness, sarcopenia, cognitive diseases, infections and inflammatory conditions [15,16,17].

However, few investigations have studied the association between carnitine levels and frailty. The aim of this study is to assess carnitine serum levels in frail older subjects considering the cognitive and physical performance.

## 2. Patients

The present cross-sectional study was based on the study data compiled from older outpatients. In total, 521 subjects (294 females and 227 males) were recruited from January 2009 to June 2018 in the Cannizzaro Hospital, Catania, Italy. All recruited subjects signed the informed consent for the study.

Inclusion Criteria were:

(1) Older than 65 years;
(2) Ability to understand and complete the questionnaires;
(3) Not suffering from severe mental or cognitive disorders and independence in terms of Activities of Daily Living (ADL) and Instrumental Activities of Daily Living (IADL).

Exclusion criteria were:

(1) Hematological diseases;
(2) Dehydration;
(3) Immunological diseases;
(4) Cardiac disease;
(5) Endocrinological disorders;
(6) Kidney, lung, liver disease;
(7) Malignancy;
(8) Treatment with corticosteroids;
(9) Assumption of L-carnitine and its derivates;
(10) Subjects in a vegan or vegetarian diet.

The patients were invited to participate in the study and gave a written consent, after being fully informed about the study and after they had completed a prepared self-administrated questionnaire. All the participants were instructed not to change their normal eating habits during the entire period of data collection.

## 3. Methods

### 3.1. Demographic Data Collection

All demographic data were collected and recorded by trained staff. Weight and height were measured without shoes. Body mass index (BMI) was calculated by the weight in kilograms divided by the height squared in meters (kg/m^2^).

Participants completed a self-reported questionnaire. Demographic factors included age, sex, residency and educational level. Lifestyle factors included physical activity and smoking status. Baseline characteristics of the patients were recorded on entry into the study. We collected information regarding the demographic profile (age, sex), liver and kidney functions (bilirubin, aspartate aminotransferase, alanine aminotransferase, alkaline phosphatase, albumin, creatinine). Other potentially confounding variables were collected and included comorbidities such as cancer, hypertension, diabetes, chronic obstructive lung disease or smoking, and eventual current procedures for cancer (any malignancy on a pathology report) (Table 1, Table 2, Table 3).

### 3.2. Laboratory Parameters

Blood samples were collected after an overnight fast and measured with an automatic biochemical analyzer to measure triglyceride serum levels, fasting plasma glucose, creatinine, urea, total cholesterol (TCH), low-density lipoprotein cholesterol (LDL-CH), high-density lipoprotein cholesterol (HDL-CH), and total bilirubin. We measured serum alanine aminotransferase (ALT) and aspartate aminotransferase (AST) and creatin-kinase (CK) using enzymatic colorimetric test.

### 3.3. L-Carnitine Determination

Patients were studied in the morning between 8:00 and 10:00 a.m. after an overnight fast. The patients were firstly asked to empty their bladder. Then, venous blood samples were drawn into tubes containing EDTA or heparin, and serum or plasma was obtained by centrifugation. A spot urine sample was obtained 10 min after the collection of the blood sample. Serum was measured immediately; plasma and urine were stored at 20 °C until analysis. The L-Carnitine concentration in plasma and urine was measured by a method described by Cederblad and Lindstedt [18] and modified by Brass and Hoppel. Plasma was treated with perchloric acid (final concentration of 3% *v/v*) and centrifuged for 2 min at 10,000× *g*. Long-chain acylcarnitines (LCACs) were measured in the pellet after alkaline hydrolysis, and free and short-chain acylcarnitines (SCACs) were measured in the supernatant fluid–the inter-assay coefficients of variation (CVs) were 3.4%, 3.8%, and 4.2%, respectively; the intra-assay CVs were 5.0%, 5.2%, and 6.0%, respectively. Addition of LCACs, together with the free and SCACs yields the total content of Acyl Carnitine (AC). No perchloric acid precipitation was performed in urine (LCACs are normally not found in urine). Free carnitine and SCACs were measured in plasma. The within-assay CVs were 4.0% and 3.8%, respectively; the between-assay CVs were 5.4% and 5.6%, respectively.

### 3.4. Frailty Score

The frailty phenotype is based on 5 predetermined criteria (involuntary weight loss, exhaustion, muscle weakness, slow gait speed and sedentary behavior).

Three or more positive criteria define the status of frailty, 1 or 2 positive criteria identify prefrailty, while the absence of positive answers indicates robustness.

Criteria of the frail phenotype are characterized by some clinical indicators, such as the unintentional weight loss, self-reported exhaustion, weakness, slow walking speed and low physical activity.

(a) Shrinking was defined as an unintentional weight loss ≥ 4.5 kg in the previous six months;
(b) Weakness was assessed by grip strength which was measured in kg with a hand-held dynamometer;
(c) Exhaustion was established based on response “no” to the question: “Do you feel full of energy?”;
(d) Slowness was established with a fast gait speed test and was measured in seconds over a six-meter course;
(e) How physical activity was determined by a self-reported response of “never” or “rarely” to the question: ”How often do you participate in physical activities?” [19].

According to these clinical criteria, 3 phenotypes have been identified:

(1) Robust: 0 criteria;
(2) Prefrail: between 1 and 2 criteria;
(3) Frail: 3 or more criteria.

### 3.5. Neuropsychological Tests

Mini Mental-Status Examination (MMSE): MMSE was used to assess cognitive functions. The MMSE score ranges between 0 and 30 [20].

Functional assessment was performed with the use of the Katz Index of Activities of Daily Living (ADLs: range 0–6). ADL questions included walking, feeling, bathing, using toilet and dressing [21]. IADL (Instrumental Activities of Daily Living) range from 0 to 8 for women and 0 to 5 for men including ability to use phone, shopping, food preparation, housekeeping, laundry, mode of transportation, responsibility for own medications, ability to handle finances [22].

Physical activity was evaluated with a 6-minute walking test. The participants walked in a corridor of known length for 6 minutes. The 6-minute walk test (6MWT) was performed in the morning in a quiet room, after an overnight fast and at a constant temperature of 22 ± 2 °C. The walking distance was the distance in meters walked by the participants in 6 minutes. The short physical performance battery (SPPB) was performed, consisting of three domains: standing balance, 4 meters walking speed and repeated chair stands [23,24,25]. Each domain contributes 0–4 points to the total score, which ranges from 0 to 12 points. Higher scores indicate a higher level of physical functioning.

### 3.6. Ethics Approval and Consent to Participate

The study protocol was approved by the Human Ethics Review Committee of Cannizzaro Hospital and a signed consent form was obtained from each patient. Study recruitment was performed in observation and respect of Helsinki Declaration. All patients gave their written informed consent for the study participation and consent for the publication. The study was approved by the Institutional Review Board (22c, g.s. 7-2-08).

### 3.7. Statistics

Data were described as mean ± standard deviation (SD) for normally distributed variables and analyzed by ordinary one-way ANOVA with Tukey’s multiple comparisons test.

Table 2 was analyzed with Fisher‘s exact test, 95% and the CI Odds Ratio (O.R.) with the Baptista-Pike method.

A *p* value < 0.05 was evaluated as statistically significant. Data were analyzed using the GraphPad Prism 8 statistical software package (8.4.2 Macintosh Version; GraphPad Software San Diego, CA, USA).

## 4. Results

The participants were classified as frail, pre-frail and robust subjects. The frail participants were 187 (105 females and 82 males), aged 74.2 ± 5.1 years; 166 were the pre-frail (95 females and 71 males), aged 75.1 ± 4.9 years and 168 were the robust subjects (94 females and 74 males), aged 75.0 ± 4.8 years.

The basal characteristic of frail, prefrail and robust patients are described in Table 1.

No significant difference between the groups were observed in education, in lifestyle and morbidity (Table 2).

No significant differences between the groups were observed also in age, systolic and diastolic pressure.

Significant differences in BMI were observed between frail vs. pre frail *p* < 0.0001 (95% CI −2.67 to −1.12), between frail vs. robust *p* < 0.0001 (95% CI −3.77 to −2.22) and between prefrail vs. robust subjects *p* = 0.0036 (95% CI −1.89 to 0.30) (Table 1).

Comparison of biochemical, metabolic and neuropsychological findings in participants.

(a) The mean serum concentrations were lower in frail elderly subjects than those in pre-frail subjects in terms of albumin *p* < 0.0001, in AST *p* = 0.003, in CK *p* < 0.0001, in ALP *p* < 0.0001, in CRP *p* < 0.0001, in TCH *p* < 0.0001, in HDL CH *p* = 0.0034 and in Triacylglycerols *p* < 0.0001, in SPPB *p* < 0.0001, in 6MWT *p* < 0.01, in MMSE *p* < 0.01 and higher in glycemia *p* < 0.0001.
(b) The mean serum concentrations were lower in frail than in robust subjects in terms of albumin (*p* < 0.0001), AST (*p* = 0.003), ALP (*p* < 0.0001), CK (*p* < 0.0001), iron (*p* < 0.0001), CRP (*p* < 0.001), in both total and HDL CH (*p* < 0.0001), and Triacylglycerols (*p* < 0.0001), in SPPB *p* < 0.001, in 6MWT *p*< 0.01, in MMSE *p* < 0.001. They were higher in urea (*p* = 0.02) and glucose (*p* = 0.016).
(c) The mean serum concentrations in prefrail subjects compared to robust elderly subjects were lower in blood glucose (*p* < 0.05), albumin (*p* < 0.0001), ALP (*p* < 0.0001), CK (*p* < 0.0001), CRP (*p* < 0.0001), TCH (*p* < 0.0001), HDL cholesterol (*p* = 0.0044), Triacylglycerols (*p*< 0.0001), in SPPB *p* < 0.001, in 6MWT *p* < 0.001.

### 4.1. Plasma Carnitine

The mean serum concentrations of total carnitine were lower in frail elderly subjects than those in prefrail (95% CI −5.87 to −1.33; *p* = 0.0006) ones, lower in frail vs. robust subjects (95% CI −10.26 to −5.74; *p* < 0.0001) and lower in prefrail vs. robust subjects (95% CI −6.73 to −2.07; *p* < 0.0001).

The mean serum concentrations of free carnitine were lower in frail elderly subjects than those in prefrail ones (95% CI −6.48 to −2.32; *p* < 0.0001), lower in frail vs. robust subjects (95% CI −9.77 to −5.63; *p* < 0.0001) and lower in prefrail vs. robust participants (95% CI −5.43 to −1.17; *p* = 0.0009).

**Figure 1:**
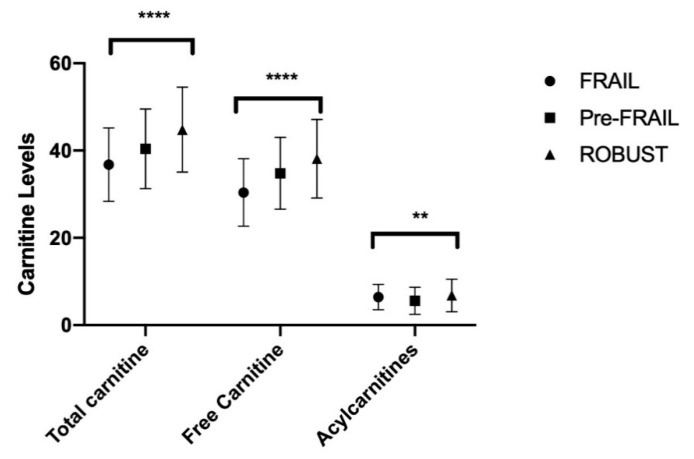
Carnitine in plasma. Summary *p* value: ** *p* < 0.005; **** *p* < 0.0001.

The mean serum concentrations of AC were higher in frail elderly subjects than those in prefrail (95% CI −0.01 to 1.61; *p* = 0.054) ones and were higher in pre-frail vs. robust subjects (95% CI −2.03 to −0.37; *p*= 0.0022) (Table 4, Figure 1).

### 4.2. Carnitine in Urine

The mean urine concentrations of total carnitine were lower in frail elderly subjects than those in prefrail (95% CI −3.86 to −0.34; *p* < 0.05) ones and were also lower in frail vs. robust subjects (95% CI −4.85 to −1.35; *p* < 0.0001).

**Figure 2:**
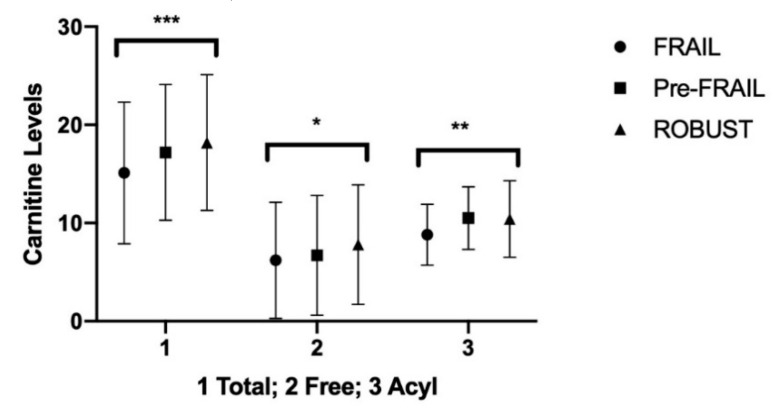
Carnitine in urine; Summary *p* value: * *p* < 0.05; ** *p* < 0.005; *** *p* < 0.001.

The mean urine concentrations of FC were lower in frail elderly vs. robust subjects (95% CI −3.10 to −0.09; *p* < 0.05). The mean urine concentrations of acyl carnitines were lower in frail elderly subjects than both those found in prefrail (95% CI −2.55 to −0.85; *p* < 0.0001) and in robust subjects (95% CI −2.45 to −0.75; *p* < 0.0001) (Table 5, Figure 2).

The data obtained showed significant correlation among levels of serum total and free carnitine and BMI (*p* < 0.01), total cholesterol (*p* < 0.01) and SPPB (*p* < 0.01).

## 5. Discussion

A low level of albumin increased markers of thrombosis as well as changes in protein related to anemia and decreased the levels of creatinine and cholesterol. These compounds have been proposed as biomarkers [26].

Aging has been associated with mitochondrial dysfunction and altered metabolic states [27].

Mitochondrial disorders affect oxidative phosphorylation, beta oxidation and energy metabolism [28,29].

In addition, mitochondria have a high concentration of 22:6*n*−3 (Docosahexaenoic Acid)-containing phospholipids and therefore these phospholipids may be needed for respiratory chain complexes [30,31].

In our study, we observed that TC serum concentration as well as the mean serum concentration of free carnitine (FC) were lower in frail subjects compared to both prefrail and robust subjects.

The carnitine pool comprises intracellular and extracellular compartments and it can be found in a free and esterified form. The various esters of carnitine with short-, medium- and long-chain fatty acids are present in biological systems.

Although almost all carnitine (99%) has an intracellular localization, the variations between serum AC and FC are an expression of intramitochondrial alterations and can reflect metabolic alterations in normal and in changed situations.

In fact, any metabolic change is accompanied by redistribution between the carnitine, the acylcarnitine and the free carnitine of the tissue involved [32].

AC is significantly altered in the serum of frail subjects versus pre-frail.

This condition suggests that an increased intramitochondrial acetyl-CoA pressure is affecting the acetate flux through carnitine acetyl transferase leading in turn to an increase in acetyl carnitine efflux at the expenses of FC [33].

Carnitine is an acyl group acceptor that facilitates mitochondrial export of excess carbons in the form of acylcarnitines.

Blood carnitine in humans remained unchanged with age in males, whereas an age-dependent increase was observed in females. These results suggest that the blood carnitine levels are maintained despite the slight decrease in the tissue of carnitine concentration. Carnitine endogenous biosynthesis is limited by availability of trimethyl-lysine, a by-product of lysosomal protein degradation [34]. Whole body carnitine homeostasis is regulated at multiple levels, including dietary intake, intestinal absorption, de novo biosynthesis and renal reabsorption [35]. Humans obtain carnitine from their diet and through endogenous biosynthesis. Dietary intake of LC in humans ranges from 1 to 15.1 mol/kg body weight/day, whereas the rate of biosynthesis is about 1–2.1 mol/kg body weight/day [36].

Serum concentrations in females tend to increase more with respect to age than in men. Instead, significant age-related changes were not observed in both genders. Instead, significant dependent increases in the serum acetyl carnitine concentrations were observed in both genders [37]. The serum carnitine concentration is a better nutritional marker that acetyl carnitine.

The serum carnitine concentrations have been found reduced in liver cirrhosis, in Crohn disease, in ulcerative colitis, in hemodialyzed patients, and during conditions of malnutrition [38,39,40,41]. In a previous study on centenarians, the administration of levocarnitine facilitated an increased capacity both in physical and in cognitive activity, reducing fatigue and improving cognitive functions. Higher physical performance and 6MWT were associated with high levels of carnitine.

Some studies have shown decreased serum FC and decreased serum acylcarnitine [42].

Carnitine insufficiency is commonly seen in dialysis patients and may be responsible for anemia, myopathy and cardiomyopathy [43,44] plasma concentration of FC is in dynamic balance with AC [45,46].

Carnitine deficiency in subjects may compromise cognitive and muscular functions in stress conditions. In subjects under fasting conditions, AC excretion in the urine increases to 78% of the excreted carnitine in the urine [47].

Carnitine deficiency develops secondary to increased urinary excretion of carnitine in the form of acyl-carnitines. Plasma concentration and urinary excretion of AC vary with physiological and pathological states, reflecting mainly the degree of acetyl-CoA synthesis. A decrease in carnitine content has been described in the brain and myocardium of aged animals [48]. An age-related decrease in carnitine content in the brain and plasma has been associated with an increase in carnitine content in the liver, possibly induced by an impaired transport from the liver to the blood in older animals [49,50].

Low carnitine levels are associated with a low mean score of MMSE and to a cognitive impairment.

The reduction in serum carnitine concentration might be detrimental for quality of life and for functional status of older subjects and might be causally related to the frailty condition in elderly subjects.

Changes in the participants’ functional status (ADL and IADL) were not observed because in the inclusion criteria, full scores of ADL and high scores of IADL were indicated. Higher physical performance and 6MWT were associated with high levels of carnitine [51].

### Limitations

The present study has several limitations. Firstly, this was a cross-sectional study, therefore the possibility of reverse causation should not be excluded.

Although carnitine is distributed mostly in skeletal muscle, we cannot measure the carnitine there because it is difficult to obtain biopsies in skeletal muscle, due to ethical reasons.

Furthermore, the causative conditions and changing patterns of carnitine levels varied from case to case and they should be detected by continuous monitoring. In addition, although we screened according to the frailty criteria, participants were, in general, in good health.

## 6. Conclusions

Currently, there are no single biomarkers that may predict the onset and the progression of frailty.

Several factors have been proposed as biomarkers of frailty such as proteins and peptides, including albumin, angiotensinogen, kinogen-1, hormones, (including testosterone, triiodothyronine, dehydroepiandrosterone sulphate), vitamin D levels, clinical markers of inflammation (including erythrocyte sedimentation, CRP, IFN alfa, Interleukin 6, white blood cell counts), or clinical markers of coagulation (including tPA, Factor VIII, D-Dimer and Fibrinogen) [52,53].

Aging-related changes in the biological system, particularly the mitochondria dysfunction, suggest that L-carnitine deficiency may be associated with aging and age-related disorders [54,55,56,57].

Further studies are needed because oral supplementation with carnitine and its derivates may reverse the age-related decline and may reduce frailty in older subjects.

Future approaches to the identification approach that includes longitudinal and interventional studies are necessary to fully elucidate these associations.

## Figures and Tables

**Table 1:** Baseline characteristics.

	(A) FRAIL		(B) Pre-FRAIL		(C) ROBUST		FRAIL vs. Pre-FRAIL (A vs. B)			FRAIL vs. ROBUST (A vs. C)			Pre-FRAIL vs. ROBUST (B vs. C)		
	*n* = 187		*n* = 166		*n* = 168										
	F = 105	M = 82	F = 95	M = 71	F = 94	M = 74									
	Mean	SD	Mean	SD	Mean	SD	95% CI	S	*p*	95% CI	S	*p*	95% CI	S	*p*
Age years	74.2	5.1	75.1	4.9	75	4.8	−2.139 to 0.3385	ns	0.2031	−2.035 to 0.4346	ns	0.2809	−1.171 to 1.371	ns	0.9813
Body Mass Index (BMI) kg/m^2^	22.8	3.1	24.7	3	25.8	3.2	−2.677 to −1.123	****	<0.0001	−3.775 to −2.225	****	<0.0001	−1.898 to −0.3022	**	0.0036
Systolic Blood Pressure (SBP) mmHg	130.2	13.1	128.2	14.2	129.1	15.8	−1.600 to 5.600	ns	0.3926	−2.489 to 4.689	ns	0.7515	−2.033 to −0.3667	ns	0.8348
Diastolic Blood Pressure (DBP) mmHg	77.2	10.1	75.8	12.4	77.1	11	−1.398 to 4.198	ns	0.468	−2.689 to 2.889	ns	0.9961	−4.171 to 1.571	ns	0.5368
Heart Rate (HR) bpm	84.1	9.7	88	10.2	87	10.8	−6.463 to −1.337	**	0.0011	−5.455 to −0.3455	*	0.0214	−1.630 to 3.630	ns	0.6445

SD: Standard Deviation; CI: Confidence Interval; S: Significance; Summary *p* value: * *p* ≤ 0.05; ** *p* < 0.005; **** *p* < 0.0001; ns: non-significant.

**Table 2:** Education, lifestyle and morbidity.

	(A) FRAIL		(B) Pre-FRAIL		(C) ROBUST		FRAIL vs. Pre-FRAIL (A vs. B)			FRAIL vs. ROBUST (A vs. C)			Pre-FRAIL vs. ROBUST (B vs. C)		
	*n* = 187		*n* = 166		*n* = 168										
	*n*	%	*n*	%	*n*	%	95% CI O.R.	S	*p*	95% CI O.R.	S	*p*	95% CI O.R.	S	*p*
No Diploma	88	47.06%	74	44.58%	75	44.64%	0.7320 to 1.673	ns	0.6694	0.7320 to 1.664	ns	0.6706	0.6441 to 1.544	ns	>0.9999
High School Dipl.	61	32.62%	58	34.94%	57	33.93%	0.5847 to 1.391	ns	0.6536	0.6115 to 1.457	ns	0.822	0.6654 to 1.645	ns	0.9084
University level	38	20.32%	34	47.73%	36	21.43%	0.5849 to 1.641	ns	>0.9999	0.5580 to 1.575	ns	0.896	0.5649 to 1.571	ns	0.8933
Smokers	96	51.34%	84	50.60%	90	53.57%	0.6835 to 1.552	ns	0.9153	0.6070 to 1.374	ns	0.7496	0.5751 to 1.367	ns	0.6614
Cerebrovascular diseases	14	7.49%	13	7.83%	13	7.74%	0.4535 to 2.044	ns	>0.9999	0.4596 to 2.070	ns	>0.9999	0.4618 to 2.222	ns	>0.9999
Heart diseases	12	6.42%	10	6.02%	11	6.55%	0.4504 to 2.524	ns	>0.9999	0.4065 to 2.159	ns	>0.9999	0.3780 to 2.127	ns	>0.9999
Joint disease	44	23.53%	40	24.10%	41	24.40%	0.5864 to 1.561	ns	0.9011	0.5793 to 1.550	ns	0.9011	0.6001 to 1.608	ns	>0.9999
Diabetes	10	5.35%	12	7.23%	10	5.95%	0.3074 to 1.725	ns	0.5132	0.3602 to 2.213	ns	0.8219	0.5226 to 2.912	ns	0.6656
Hypertension	20	10.70%	18	10.84%	16	9.52%	0.5092 to 1.947	ns	>0.9999	0.5720 to 2.207	ns	0.7289	0.5524 to 2.301	ns	0.7206

SD: Standard Deviation; CI: Confidence Interval; O.R.: Odds Ratio; S: Significance. Summary *p* value: ns: non-significant.

**Table 3:** Biochemical and metabolic parameters.

	FRAIL		Pre-FRAIL		ROBUST		FRAIL vs. Pre-FRAIL		FRAIL vs. ROBUST		Pre-FRAIL vs. ROBUST	
	187		166		168							
	Mean	SD	Mean	SD	Mean	SD	*p*	S	*p*	S	*p*	S
Urea [mg/dL]	43.2	8.7	41.8	9.2	40.7	8.9	0.3059	ns	0.0235	*	0.4984	ns
Glucose [mg/dL]	88.1	10.2	82.5	9.7	85.2	9.8	<0.0001	****	0.0169	*	0.035	*
Albumin [g/dL]	3.67	0.36	3.96	0.44	4.1	0.31	<0.0001	****	<0.0001	****	0.0019	**
Creatinine [mg/dL]	0.9	0.34	0.87	0.44	0.97	0.36	0.7405	ns	0.1951	ns	0.0441	*
AST [IU/L]	38.2	5.1	36.4	5.4	37.2	5.2	0.0038	**	0.1709	ns	0.3427	ns
ALT [IU/L]	30.1	5.4	30.6	5.9	31.8	5.1	0.6678	ns	0.0101	*	0.1123	ns
CK [IU/L]	33.2	10.1	39.1	10.4	44.2	10.7	<0.0001	****	<0.0001	****	<0.0001	****
ALP [IU/L]	104.2	8.7	112.4	7.9	101.9	8.7	<0.0001	****	0.029	*	<0.0001	****
CRP [mg/dL]	2.1	0.5	2.4	0.44	2.96	0.67	<0.0001	****	<0.0001	****	<0.0001	****
BT [mg/dL]	0.94	0.2	0.9	0.3	0.91	0.28	0.3236	ns	0.527	ns	0.9349	ns
T CH [mg/dL]	151.2	38	168	40	187	37	0.0001	***	<0.0001	****	<0.0001	****
HDL CH [mg/dL]	38.1	3.1	39.2	3.7	40.3	2.6	0.0034	**	<0.0001	****	0.0044	**
Triacylglycerols [mg/dL]	138.4	2.7	149.2	23.8	167.2	31.2	<0.0001	****	<0.0001	****	<0.0001	****
MMSE score	22.4	2.7	23.6	3.1	23.9	4.1	0.0023	**	<0.0001	****	0.689	ns
SPPB	7.2	1.4	8.4	1.2	9.8	0.6	<0.0001	****	<0.0001	****	<0.0001	****
6MWT [m]	7.4	3.7	9.2	6.2	12.4	4.44	0.0016	**	<0.0001	****	<0.0001	****

CK: creatine kinase; AST: aspartate aminotransferase; ALT: alanine aminotransferase; ALP: alkaline phosphatase; CRP: C reactive protein; T CH: total cholesterol; HDL CH: HDL cholesterol; MMSE: mini-mental state examination; SPPB: short physical performance battery; 6MWT: 6-minute walk test; SD: Standard Deviation; S: Significance; Summary *p* value: * *p* ≤ 0.05; ** *p* < 0.005; *** *p* < 0.001; **** *p* < 0.0001; ns: non-significant.

**Table 4:** Carnitine in the plasma.

PLASMA	(A) FRAIL		(B) Pre-FRAIL		(C) ROBUST		FRAIL vs. Pre-FRAIL (A vs. B)			FRAIL vs. ROBUST (A vs. C)			Pre-FRAIL vs. ROBUST (B vs. C)		
	*n* = 187		*n* = 166		*n* = 168										
	Mean	SD	Mean	SD	Mean	SD	95% CI	S	*p*	95% CI	S	*p*	95% CI	S	*p*
TC	36.8	8.4	40.4	9.1	44.8	9.7	−5.870 to −1.330	***	0.0006	−10.26 to −5.737	****	<0.0001	−6.730 to −2.070	****	<0.0001
FC	30.4	7.7	34.8	8.2	38.1	9	−6.479 to −2.321	****	<0.0001	−9.773 to −5.627	****	<0.0001	−5.434 to −1.166	***	0.0009
AC	6.4	2.9	5.6	3.1	6.8	3.7	−0.01198 to 1.612	ns	0.0545	−1.209 to 0.4094	ns	0.4768	−2.033 to −0.3667	**	0.0022

TC: total carnitine; FC: free carnitine; AC: acylcarnitine; SD: Standard Deviation; CI: Confidence Interval; S: Significance; Summary *p* value: ** *p* < 0.005; *** *p* < 0.001; **** *p* < 0.0001; ns: non-significant.

**Table 5:** Carnitine in the urine.

URINE	FRAIL		Pre-FRAIL		ROBUST		FRAIL vs. Pre-FRAIL (A vs. B)			FRAIL vs. ROBUST (A vs. C)			Pre-FRAIL vs. ROBUST (B vs. C)		
	*n*= 187		*n* = 166		*n* = 168										
	Mean	SD	Mean	SD	Mean	SD	95% CI	S	*p*	95% CI	S	*p*	95% CI	S	*p*
TC	15.1	7.2	17.2	6.9	18.2	6.9	−3.857 to −0.3431	*	0.0142	−4.851 to −1.349	***	0.0001	−2.803 to 0.8030	ns	0.3938
FC	6.2	5.9	6.7	6.1	7.8	6.1	−2.011 to 1.011	ns	0.7169	−3.106 to −0.09362	*	0.0343	−2.651 to 0.4508	ns	0.2189
AC	8.8	3.1	10.5	3.2	10.4	3.9	−2.554 to −0.8457	****	<0.0001	−2.452 to −0.7484	****	<0.0001	−0.7767 to 0.9767	ns	0.9612

TC: total carnitine; FC: free carnitine; AC: acylcarnitine; Summary *p* value: * *p* ≤ 0.05; *** *p* < 0.001; **** *p* < 0.0001; ns: non-significant.

## Data Availability

The datasets used and/or analyzed during the current study are available from the corresponding author on a reasonable request.
